# Therapeutic Efficacy of Quercetin and Its Nanoformulation Both the Mono- or Combination Therapies in the Management of Cancer: An Update with Molecular Mechanisms

**DOI:** 10.1155/2024/5594462

**Published:** 2024-10-01

**Authors:** Tanzila Akter Eity, Md. Shimul Bhuia, Raihan Chowdhury, Shakil Ahmmed, Salehin Sheikh, Rima Akter, Muhammad Torequl Islam

**Affiliations:** ^1^ Department of Biotechnology and Genetic Engineering Bangabandhu Sheikh Mujibur Rahman Science and Technology University, Gopalganj, Gopalganj 8100, Bangladesh; ^2^ Phytochemistry and Biodiversity Research Laboratory BioLuster Research Center Ltd., Gopalganj, Gopalganj 8100, Bangladesh; ^3^ Department of Pharmacy Bangabandhu Sheikh Mujibur Rahman Science and Technology University, Gopalganj, Gopalganj 8100, Bangladesh; ^4^ Department of Biochemistry and Molecular Biology Bangladesh Agricultural University, Mymensingh 2202, Bangladesh; ^5^ Biotechnology and Genetic Engineering Discipline Khulna University, Khulna 9208, Bangladesh; ^6^ Pharmacy Discipline Khulna University, Khulna 9208, Bangladesh

## Abstract

Quercetin, a major representative of the flavonol subclass found abundantly in almost all edible vegetables and fruits, showed remarkable therapeutic properties and was beneficial in numerous degenerative diseases by preventing lipid peroxidation. Quercetin is beneficial in different diseases, such as atherosclerosis and chronic inflammation. This study aims to find out the anticancer activities of quercetin and to determine different mechanisms and pathways which are responsible for the anticancer effect. It also revealed the biopharmaceutical, toxicological characteristics, and clinical utilization of quercetin to evaluate its suitability for further investigations as a reliable anticancer drug. All of the relevant data concerning this compound with cancer was collected using different scientific search engines, including PubMed, Springer Link, Wiley Online, Web of Science, SciFinder, ScienceDirect, and Google Scholar. This review demonstrated that quercetin showed strong anticancer properties, including apoptosis, inhibition of cell proliferation, autophagy, cell cycle arrest, inhibition of angiogenesis, and inhibition of invasion and migration against various types of cancer. Findings also revealed that quercetin could significantly moderate and regulate different pathways, including PI3K/AKT-mTORC1 pathway, JAK/STAT signaling system, MAPK signaling pathway, MMP signaling pathway, NF-*κ*B pathway, and p-Camk2/p-DRP1 pathway. However, this study found that quercetin showed poor oral bioavailability due to reduced absorption; this limitation is overcome by applying nanotechnology (nanoformulation of quercetin). Moreover, different investigations revealed that quercetin expressed no toxic effect in the investigated subjects. Based on the view of these findings, it is demonstrated that quercetin might be considered a reliable chemotherapeutic drug candidate in the treatment of different cancers. However, more clinical studies are suggested to establish the proper therapeutic efficacy, safety, and human dose.

## 1. Introduction

Cancer refers to the condition when tumor cells can proliferate and invade other tissues around the tumor cell by halting tumor-suppressing genes and upregulating oncogenes, genomic instability, and intracellular signaling cascades [1]. Cancer is a significant global health burden. Cancer is the second leading cause of death in many nations, after heart disease. Every year, a significant number of people, approximately ten million, get cancer globally and almost fifty percent of these patients ultimately die from the disease [2]. According to the survey by the International Agency for Research on Cancer (IARC), 20 million new cancer patients will be diagnosed and almost 9.7 million patients died of cancer in 2022. By 2030, it is projected that there will be a significant increase in the current burden of cancer with an annual occurrence of 22 million new cancer cases and 13 million deaths due to cancer [3, 4]. In Bangladesh, the number of new cancer cases is 1,67,256 and the number of deaths is 1,16,598 due to cancer in 2022 [5].

In developing and low-income countries, multiple aspects of people's lifestyles are the major causes of cancer including smoking, low fruit and vegetable intake, sexual transmission of human papillomavirus, and alcohol drinking. On the other hand, in highly developed countries, significant causes of cancer are drinking alcohol, smoking, overweight, and obesity [6]. IARC also investigated several factors associated with cancer development such as salted fish, sunlight, pharmaceuticals, hormones, tobacco, parasites, bacteria, fungi, herbs, and wood dust. Different causes of cancer were also identified by the World Cancer Research Fund and the American Institute for Cancer Research which include red meat, beta carotene, low fiber diets, processed meats, not breastfeeding, increased adult height, and sedentary lifestyles [7]. Cancer's symptoms and risks can affect a patient's quality life. Treatment, disease acceptance, organ displacement, psychological distress, pain and duration of disease, etc., could affect a patient's quality of life [8]. Numerous transcription factors and proteins (NF-*κ*B, AP-1 and STAT3, cyclooxygenase-2, interleukin-1, interleukin-6, chemokines, tumor necrosis factor, 5 lipoxygenase, matrix metalloproteases, vascular endothelial growth factor, and adhesion molecules) cause inflammation in cancer [9]. There are different conventional treatment strategies like chemotherapy, radiotherapy, and surgery, but they have several side effects including they also destroy the healthy cells, nausea, fatigue, hair loss, vomiting, etc., and they can also cause death in different cases [10]. Therefore, it is required to apply alternate concepts or approaches for the prevention of cancer [11]. In recent times, several advanced approaches have been made to avoid these side effects including ablation therapy, targeted therapy, nanoparticles, natural antioxidants, natural compounds, and ferroptosis-based therapy [12].

From early human history, natural products have been studied and used to treat disease [13]. The use of natural products, extracted from plants, fungi, and herbs as therapeutic compounds is considered as a significant precursor to modern medicine due to their limited side effects, reliable safety records, and cost-effectiveness. These natural compounds have two applications, being used as cancer therapeutics and chemopreventive chemical substances [14–16]. Besides, natural products have various significant biochemical properties and have shown remarkable anticancer activity for more than fifty years [17].

Quercetin, a unique bioflavonoid, chemically known as 3, 3′, 4′, 5, 7-pentahydroxyflvanone (synonym 2-(3,4-dihydroxyphenyl)-3,5,7-trihydroxy- 4H-chromen-4-one), found widely in different food products including berries, apples, cauliflower, tea, cabbage, nuts, onions, broccoli, and berries, which has numerous pharmacological properties including anticancer activities, antiaging, anti-inflammatory [18, 19], antiviral activities, as well as reducing lipid peroxidation, platelet aggregation, and capillary permeability [19, 20]. It has more biological properties, including antioxidant [21], antifungal [22], anticarcinogenic, hepatoprotective [23], and cytotoxic activity [24]. It is demonstrated to accumulate in the lungs, liver, kidneys, and small intestines, and it is extracted through the renal, fecal, and respiratory systems [25].

Different *in vitro* and some animal models studies have shown that quercetin could diminish the growth of cancer cells such as breast, bladder, colon, colorectal, prostate, and lung cancer cells [26–29]. Moreover, quercetin has significant antioxidant properties which can prevent reactive oxygen species (ROS) and trigger DNA damage and also mutational changes [30]. Different clinical and preclinical studies showed that quercetin could alleviate cell division, the invasion, and migration of different cancer cells by various types of mechanisms and pathways like apoptosis, cytotoxicity, AKT pathways, JAK/STAT pathways, ferroptosis, angiogenesis, etc. [31–33].

The novelty of this study from other previous studies is that our study describes the pharmacokinetic properties and anticancer activity of quercetin in monotherapy and nanotherapy, along with different anticancer mechanisms in various types of cancer cells. This study also demonstrated the toxicological profile of quercetin and the role of quercetin in immunotherapy. We found that different *in vivo* and *in silico* studies indicated the synergistic potential of combination and nanotherapy. Complexities with other drugs or nanoparticles significantly enhanced the bioavailability, solubility, and potency of quercetin, which could improve the potentiality of quercetin to develop into a reliable drug for the treatment of cancer. The main aim of this review is to summarize the biopharmaceutical properties of quercetin. Our focus will be on discussing the anticancer activities of quercetin as well as its biological sources. Moreover, our objective is to determine different mechanisms and pathways which are significant for anticancer activities.

## 2. Methodology

### 2.1. Literature Search Stratagem

The data were collected (up to August 16, 2024) by searching electronic databases such as PubMed, ScienceDirect, Springer Link, Scopus, Wiley Online, Web of Science, ResearchGate, and Google Scholar with the terms “Quercetin,” then paired with “Cancer,” “Tumor,” “Pathophysiology of cancer,” “Anticancer activity,” “Antiproliferation activity,” “Apoptotic effect,” “Oxidative stress,” “Protective effect,” “Cytotoxic activity,” “Genotoxic activity,” “Carcinogenesis,” “Anti-angiogenic effect,” “Antitumor activity,” “Human cancer,” “Biological activities,” “Biological evaluation,” “Chemical features,” “Pharmacokinetics,” “Biopharmaceutics,” “Medicinal use,” “Pharmacology,” “Pharmacological effects,” “Pharmacological activities,” “*In vivo* studies,” or “*In vitro* studies.” No language restrictions were imposed. The studies were thoroughly assessed, with information on the sources, dose, concentration, test system, hypothesized anticancer effect mechanism, and overall conclusion provided. The following are the criteria for inclusion and exclusion.

### 2.2. Inclusion and Exclusion Criteria

Inclusion criteria were defined as follows: (a) studies performed in different laboratory animals, humans, and their derived tissues or cells; (b) studies of the anticancer activities of quercetin; (c) studies with quercetin in combination with other molecules; (d) studies with or without hypothesized mechanisms of action; (e) studies with the physical and chemical characteristics of quercetin; (f) studies with the biopharmaceutical profiles of quercetin or its preparations; (g) studies with the toxicological profile of quercetin; (h) studies of clinical investigation of quercetin; and (i) studies of the anticancer properties of quercetin investigated up to date 2024.

Exclusion criteria were defined as follows: (i) Studies exhibited duplicate data and/or titles and abstracts that did not meet the inclusion criteria; (ii) quercetin, in conjunction with other studies, sheds light on the current issue; (iii) papers written in languages other than English; (iv) studies do not have complete written content accessible; and (v) case reports, letters, editorials, and commentaries.

### 2.3. Biological Sources of Quercetin

Medicinal plants are globally recognized as valuable resources for the exploration of new pharmaceuticals [34, 35]. Traditional herbal remedies have long been an integral part of healthcare systems across the world, helping with a wide range of acute and chronic illnesses with little to no side effects. A wide range of medical conditions, including cancer, tuberculosis, diabetes, wound healing, heart disease, pharyngitis, asthma, hypertension, and many more, have traditionally been treated using herbal remedies [36, 37]. Seventy to ninety-five percent of people in underdeveloped nations use medicinal plants as their main source of treatment, according to the World Health Organization (WHO). Despite their extensive usage, only a few medicinal plants have had their phytochemical and phytopharmacological characteristics studied in order to determine their possible therapeutic effects [38].

One of the several flavonoid compounds found naturally in plants, quercetin, has many medicinal uses [39]. Several plant's aerial parts, seeds, roots, and leaves contain the chemical source, as described in published research. The botanical origins of this phytochemical are shown in Table 1.

### 2.4. Pharmacokinetics

Pharmacokinetics (PK) is a significant concept that determines the final clinical success or failure of a drug to treat a disease [61]. To determine the exact concentrations and the time course for a drug to act properly, different pharmacological characteristics like clearance, biological half-life, and bioavailability are need to be taken into consideration. These factors are important to characterize the effects of disease with respect to drug absorption, distribution, excretion, and metabolism (ADME) in each patient [62, 63]. Bioavailability is a major pharmacokinetic parameter which demonstrates the concentration of a nonvascular drug which can enter the systemic circulation through a nonvascular route. The adsorption rate is also another important PK parameter that is evaluated by calculating the value of *C*_max_ and *T*_max_ [64]. However, in new drug discovery and development, ADME effects of drugs are a significant consideration. Besides, inappropriate PK often creates challenges during the drug development process. Moreover, the main reasons for costly failures in developing medications during the later stages were the unfavorable pharmacokinetic characteristics [65, 66]. Different studies demonstrated that low PK and bioavailability are the major causes of medication failure, which represent approximately 40% of the causes [67, 68].

Quercetin (C_15_H_10_O_7_) is a lipophilic compound (yellow crystalline powder) with a high molecular mass, density, and melting point of 302.236 g/mol, 1.799 g/cm^3^, and 316°C, respectively [69]. The pharmacokinetic properties of quercetin are poor solubility, low bioavailability, poor permeability, and instability [70]. Moreover, quercetin has very poor water solubility. However, different studies demonstrated that the water solubility of quercetin could increase rapidly and significantly with temperature. The water solubility of quercetin varied from 0.00215 g/L at 25°C to 0.665 g/L at 140°C [25, 71]. Furthermore, due to the macronutrient absorption, the oral bioavailability of quercetin is very poor after a single oral dose [72].

So, to develop quercetin as a potential drug, it is necessary to improve its pharmacokinetic properties. In order to enhance the bioavailability of quercetin, various strategies have been employed, including the application of potential drug delivery methods such as inclusion complexes, liposomes, nanoparticles, or micelles, which have been demonstrated to increase efficacy, stability, solubility, and bioavailability [70, 73]. For instance, quercetin nanoparticles have shown numerous advantages including a high degree of encapsulation efficiency, high stability, prolonged release, circulation for a long time, accumulation effectively at tumor sites, and improved therapeutic efficacy [74].

**Figure 1:**
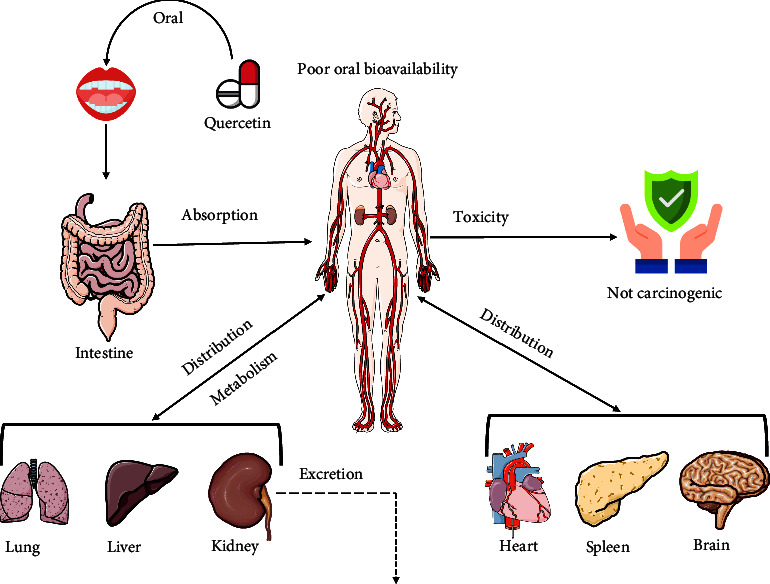
Pharmacokinetics and bioavailability of quercetin.

Quercetin also has a low adsorption quality in the gut and stomach after oral intake [75]. The primary adsorption site of quercetin is the small intestine where it enters through the sodium-dependent glucose cotransporters (SGLTs) expressed on the apical membrane of intestinal epithelial cells [76, 77]. After intestinal absorption, quercetin undergoes phase II metabolism which includes different mechanisms catalyzed by sulfotransferases (SULTs), uridine-5′-diphosphate glucuronosyltransferases (UGTs), and catechol-*O*-methyl-transferases (COMTs) and ejected through bile [78]. Quercetin is highly unstable in alkaline conditions [75]. Quercetin remarkably accumulates in the lungs, liver, kidneys, and small intestines at high concentrations and in the brain, heart, and spleen at low concentrations. The brain contains a low amount of quercetin due to the presence of a specific brain transporter for quercetin. However, the excretion of quercetin occurs through the renal, fecal, and respiratory systems, and it has no carcinogenic effects [25, 79]. The pharmacokinetic activities and bioavailability of quercetin are depicted in Figure 1.

### 2.5. Anticancer Activity: Underlying Molecular Mechanistic Analysis

#### 2.5.1. Induction of Oxidative Stress

The term “oxidative stress” refers to the relative abundance of reactive oxygen species (ROS) along with antioxidants, which have a remarkable role in determining the ultimate fate of cells. It has been associated with numerous conditions, including diabetes, cancer, heart disease, and neurological disorders [14, 80]. Oxidative stress is a key factor for the development and progression of tumors, but it is also commonly found during programmed cell death when cells are exposed to different anticancer drug treatments [81]. Reactive species are mainly of four types, including ROS, reactive nitrogen species (RNS), reactive sulfur species (RSS), and reactive chloride species (RCS) which are produced by oxidative metabolism [82]. From these types, ROS are produced in abundance. The extremely high ROS concentrations are cytotoxic, which can reduce cell defense mechanisms like antioxidants [83, 84]. High ROS levels can also activate tyrosine kinases to dissociate Nrf2: Keap1 complex which is responsible for inducing apoptosis [85].

**Figure 2:**
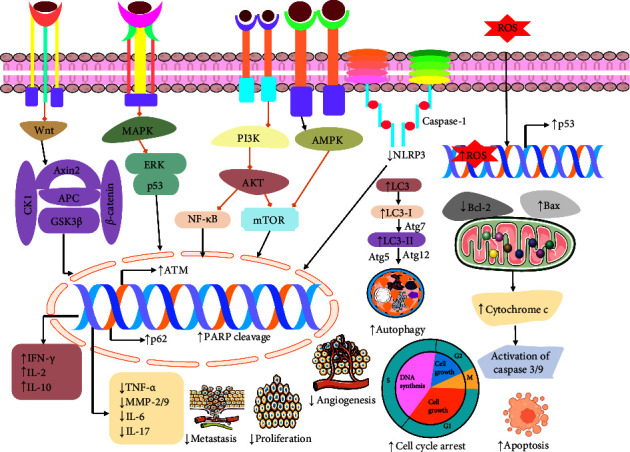
Estimated possible anticancer mechanism of quercetin involving various cellular pathways. ASC: caspase recruitment domain; NLRP3: NLR family pyrin domain containing 3; PARP: poly (ADP-ribose) polymerase; Caspase: cysteine-aspartic acid protease; MEK: mitogen-activated extracellular signal-regulated kinase; Bax: BcL2-associated X protein; Bcl-2: B-cell lymphoma; LC3-II: microtubule-associated protein 1A/1B-light chain 3; p62: ubiquitin-binding protein p62; AKT: protein kinase B; mTOR: mammalian (or mechanistic) target of rapamycin; MMP2 and MMP9: matrix metalloproteinase-2 and matrix metalloproteinase-9; PI3K: phosphoinositide 3-kinase; Beclin1: protein that regulated autophagy; IL-2: interleukin-2; IFN-*γ*: interferon-gamma; TNF-*α*: tumor necrosis factor alpha; TNF-*β*: tumor necrosis factor beta; Atg 5, 7, and 12: autophagy protein 5, 7, and 12; AMPK: AMP-activated protein kinase; NF- *κ*B: nuclear factor-*κ*B; EGRF: epidermal growth factor receptor.

A study conducted by Wu et al. [86] revealed that quercetin could induce oxidative stress in breast cancer cells (MCF-7 BC) by elevating the level of ROS in cellular mitochondria, which also induced apoptosis consequently at concentrations of 100 *μ*M [86]. Another study carried out by Wang and his team members showed that quercetin could trigger oxidative stress in colorectal cancer cells (HepG2, Hep3B, MDA-MB-231, Atg7-WT, and Atg7-KO MEF) by activating lipid peroxidation and increasing ROS levels at a 50 *μ*M concentration [87]. In gastric cancer cells (AGS and GES-1), quercetin could elevate the ROS levels in mitochondria and trigger cell death at 40 *μ*M. It also decreased the expression of NRF2 and induced oxidative stress in cancer cells [88]. Another study also found that quercetin could remarkably elevate oxidative stress in lung cancer cells by increasing ROS concentrations and DNA damage to induce cell death [89]. Ward et al. [28] investigated the capability of quercetin to induce oxidative stress in prostate cancer cells with mutated p53 (LNCaP, DU-145) and PC-3 cells via increasing ROS levels and mitogen-activated extracellular signal-regulated kinase (MEK) at the dose of 40 *μ*M [28]. Wang and his colleagues revealed that quercetin could cause oxidative stress in glioblastoma cancer cells (T98g) by increasing the expression of ROS [90]. So, the overall findings of different studies suggest that quercetin conducts anticancer activity by inducing oxidative stress in cancer cells. A brief overview of pathophysiological events including oxidative stress, apoptosis, antiangiogenesis, cell cycle arrest, autophagy, and the antiproliferative effects of quercetin are shown in Figure 2.

#### 2.5.2. Cell Cycle Arrest

Cell cycle arrest refers to a pause in the cell cycle when it declines to engage in activities related to replication and division. This cycle is one of the most crucial processes in a living cell which is strictly regulated and controlled by many mechanisms to prevent parent cell abnormalities from transferring to the daughter cell. Disrupting such systems of regulation is part of cancer pathogenesis [91, 92]. Cell cycle arrest commonly occurs at the G1/S or G2/M borders. When the regulation of checkpoint arrest is disrupted, cellular damage does not hinder the initiation of the S phase or mitosis. Consequently, inhibiting these stages leads to aberrant responses as a consequence of cellular damage [93, 94]. Retinoblastoma protein RB and the transcription factor p53 are key tumor-suppressing proteins that mainly contribute to inducing cell death and cell cycle arrest. Both proteins have essential functions in controlling the cell division cycle [95]. Through the selective targeting of specific proteins, several medications against cancer hinder the transition of cells from a single phase to another in the cell cycle, resulting in the accumulation of cancer cells at a given stage. The cell cycle is halted, hence impeding the proliferation of cancer cells into tumors and metastasizing to other regions of the body [96, 97].

Studies revealed that quercetin (10−80 *μ*M) had an anticancer effect, including its ability to arrest the cell cycle in T24 bladder cancer cells [26]. Similar promising activity was reported in breast cancer MCF-7 cells which was arresting the cell cycle at the S phase by quercetin (100 *μ*M) [86]. According to a study by García-Gutiérrez et al. [32], quercetin also showed anticancer effects in colon cancer HT-29 cells exposed to BPA, including the ability of cell cycle arrest in the G0/G1 [32]. Additionally, quercetin exhibited a cell cycle arrest in colon cancer cells (HCT 116, COLO 320, and COLO 205) [31]. A study showed that quercetin arrests the cell cycle in esophageal carcinoma cells and downregulates cell proliferation, invasion, and clonal formation [98]. Yang et al. [89] and his team investigated quercetin-induced cell cycle arrest, especially S-phase cell cycle arrest [89]. Another investigation demonstrated that quercetin is also able to arrest the G0/G1 phase and the G2/M phase in the cell cycle of breast cancer, hepatocellular carcinoma, cervical carcinoma, and human ovarian carcinoma in MCF-7, SMMC-7721, HeLa, and SKOV3 cell lines [39]. A study conducted by Hisaka et al. [99] revealed that quercetin (100 *μ*M) showed cell cycle arresting properties in KIM1, KYN-2, KYN-3, HAK-1B, HAK-2, HAK-5, HAK-6, KMCH-1 and KMCH-2 cells [99]. Son and his colleagues demonstrated that quercetin-induced cell cycle arrest specially in G1 phase resulted in reduced cell proliferation in oral squamous cell carcinomas cells (YD10B and YD38) [100]. A study revealed that quercetin (50 *μ*g/mL) suppressed malignant melanoma B16 murine melanoma cell proliferation and cell viability by arresting the cell cycle in the S and G2/M stages [101]. Another investigation demonstrated that quercetin has an anticancer effect in glioblastoma cancer cells (T98g) resulting in induced arrested cells in the S-phase cell cycle by decreasing the growth and migration of cells [90]. Song and his team exhibited that quercetin had an anticancer effect in intrahepatic cholangiocarcinoma ICC cell proliferation and survival, and invasion was suppressed through cell cycle arrest at the G1 phase [102]. All these findings demonstrated the ability of quercetin to block cell cycle progression (Figure 2).

### 2.6. Ferroptosis Cell Death

Unlike conventional apoptosis and necrosis, ferroptosis is a recently discovered sort of cell death associated with the formation of iron-dependent lipid peroxide. It is characterized by different cytological properties, including a reduction in cell volume and diminished mitochondrial cristae, a fractured outer mitochondrial membrane. It differs from apoptosis and necrosis on the basis of morphology, biochemistry, and genetics [103, 104]. Ferroptosis can be triggered by ROS accumulation and lipid peroxidation. Polyunsaturated fatty acid-containing phospholipids associated with lipid peroxidation are induced by ACSL4, LPCAT3, ALOXs, or POR enzymes. Different pathways related to autophagy including the mTOR/S6KP70 pathway and the NF-*κ*B pathway also induce ferroptosis by increasing iron concentration [105].

**Figure 3:**
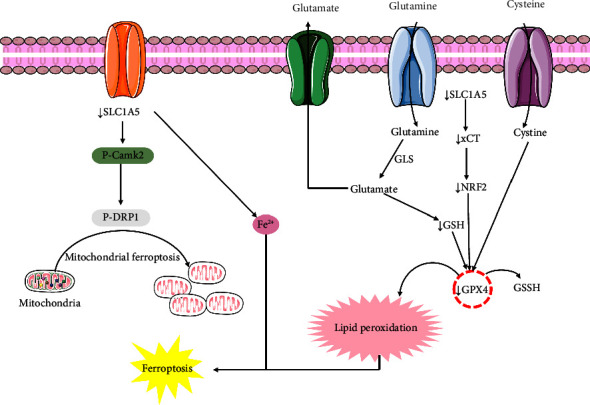
Mechanism of action of quercetin in the ferroptosis signaling pathway. SLC1A5: solute-linked carrier family A1 member 5; NRF2: nuclear factor erythroid 2 (NF-E2)-related factor 2; GSH: glutathione; GPX4: glutathione peroxidase 4; p-DRP1: dynamin-related protein 1.

A recent study carried out by Zhu and his colleagues revealed that quercetin could induce ferroptosis cell death in oral squamous cell carcinoma cells by triggering mTOR/S6KP70 and lipid peroxidation. Quercetin could also knockdown the levels of GSH and SLC7A11 [106]. Another study found the ferroptosis activity of quercetin in breast cancer cells (MCF-7 and MDA-231) via increasing the accumulation of iron, malondialdehyde (MDA), carbonylation protein (CFP), ferric ions, TFEB, and LAMP-1 at the concentrations of 0.1, 1, and 10 *μ*M [107]. Wang and his team members have investigated that quercetin could stimulate ferroptosis in colorectal cancer cells (HepG2, Hep3B, MDA-MB-231, Atg7-WT, and Atg7-KO MEF) through triggering lysosomal activation, nuclear translocation of EB, TFEB, ferritin degradation, free iron release, ROS, lipid peroxidation, Bid, cleavage of PARP, and caspase-9. The study also showed that quercetin could halt mTOR pathways to stimulate ferroptosis [87]. The upregulation of ROS levels and downregulation of the expression of SLC1A5, NRF2, xCT, and GPX4 by quercetin result in ferroptosis in gastric cancer cell lines (GC cells, AGS, and GES-1) at the dose of 40 *μ*M [88]. There is also another study that showed that quercetin could induce ferroptosis in gastric cancer cells (AGS and MKN45) by diminishing tumor volume, GSH, MDA, ROS, Beclin1, and LC3B which alternatively trigger apoptosis [108]. In intrahepatic cholangiocarcinoma cells, quercetin showed a remarkable role in inducing ferroptosis by diminishing NF-*κ*B concentrations [102]. From these investigations, it is clear that ferroptosis plays an unignorable role in the treatment of different cancer cells by halting their growth and proliferation. The mechanism of action of quercetin in the ferroptosis signaling pathway is displayed in Figure 3.

### 2.7. Apoptotic Cell Death

Apoptosis, also called programmed cell death, defines different physiological characteristics, including cell shrinkage, membrane blebbing, chromatin condensation, and nuclear fragmentation [67, 109]. The process of programmed cell death depends on various proteins and pathways. Apoptotic proteins such as Bcl-2, Bax, MCL-1, Bcl-w, caspase-3, caspase-6, caspase-7, caspase-8, and caspase-9 and BFL-1/A1 induce apoptosis via different mechanisms [110]. Numerous pathways, including the MPK pathway, the EGFR and ERK pathway, the PI3K/AKT-mTORC1 pathway, and the NF-*κ*B pathway, could stimulate apoptosis [111]. Moderation of apoptotic proteins and pathways leads to the proliferation and growth of cancer cells [112]. Different studies suggested that the moderation of the apoptotic protein and pathways might be a great target for therapeutic drugs to treat cancer by triggering programmed cell death [113].

Research findings showed that quercetin (10−80 *μ*M) induced apoptosis in bladder cancer cells (T24) via increasing cytotoxicity and decreasing cytoplasmic retraction and membrane condensation [26]. Another study conducted by Wu et al. [114] revealed quercetin (100 *μ*M) mediated apoptosis cell death in breast cancer (MCF-7 cells) by elevating cytotoxicity, ROS, and oxidative stress [86]. A study demonstrated that quercetin could induce apoptosis in breast cancer cells via stimulating the EGFR and ERK pathways [115]. Tezerji et al. [33] found quercetin (10 mg/kg) mediated apoptosis cell death in colon cancer by increasing caspase-3 and decreasing beta-catenin and Bcl-2 proteins [33]. A study conducted by García-Gutiérrez et al. [32] showed that quercetin (160.63 *μ*M) induced apoptosis in colon cancer (HT-29 cells exposed to BPA) via upregulating *ESR2* and *GPR30* genes, cytotoxicity, and downregulating cell viability [32]. A study also demonstrated that quercetin in colon cancer (HCT 116, COLO 320, and COLO 205 cells) induced apoptosis by increasing Sirtuin-6 and Klotho, cell cycle arrest, and decreasing proteasome 20S [31]. Özsoy and his colleagues demonstrated that quercetin at a concentration of 25 *μ*g/ml quercetin for 48 hours caused cell death in colon cancer (Colo-320 and Colo-741 cell lines) through upregulating p16, lamin B1 and cyclin B1, senescence, cytotoxicity, Bax, and cleaved caspase-3 and downregulating cell growth and Bcl-2 [116]. In the context of colorectal cancer in HepG2, Hep3B, MDA-MB-231, Atg7-WT, Atg7-KO MEF, and HCT116 cells, quercetin (50 *μ*M for 24 h) was investigated and found to be responsible for the apoptotic cell death via upregulating lysosomal activation, nuclear translocation of EB and transcriptional activation of lysosomal genes, cytotoxicity, TFEB, ferritin degradation, free iron release, ROS, lipid peroxidation, Bid, cleavage of PARP, and caspase-9 and downregulating mTOR, p53, PI3K/AKT-mTORC1 pathway, and MMP [87]. A study revealed that quercetin induced apoptosis in gastric cancer (AGS and MKN45 cells) by increasing ferroptosis, autophagy, and decreasing tumor volume, GSH, MDA, and ROS, Beclin1, and LC3B [108]. Guo and his team investigated that quercetin is responsible for the apoptotic cell death in lung cancer cells (A549 and H1299) at different concentrations of 12.5, 25, 50, and 100 *μ*M of for 24 hours via upregulating autophagy, LC3-II, Beclin1, Atg5, Atg7, and Atg12, SIRT1 protein, pAMPK-AMPK, cleaved caspase-3 protein, Bax, cytotoxicity, and downregulating Bcl-2 levels [27]. The apoptosis effect of quercetin was confirmed in lung cancer by several studies where quercetin showed a positive effect on apoptosis by upregulating ROS, caspase-2 and caspase-3, DNA damage, S-phase cell cycle arrest, and ATM [89]. Ward and his team revealed that quercetin (40 *μ*M) induced apoptosis in prostate cancer cells (LNCaP, DU-145 with mutated p53, and PC-3) by increasing Raf, MEK, ROS, and Bax levels and decreasing cell viability, the AKT pathway, NF-*κ*B pathway, and Bcl-2 [28]. Another study revealed that quercetin showed a positive effect on apoptosis in different cancers, including breast cancer, hepatocellular carcinoma, cervical carcinoma, and human ovarian carcinoma cell lines (MCF-7, SMMC-7721, HeLa, and SKOV3) via Bax and downregulating Bcl-2 [39]. Quercetin-induced apoptosis in lung cancer cells (BEAS-2B, A549, and H1299) through increasing caspase-3, Bax, DNA damage, p-CDK1, and ferroptosis as well as decreasing SIRT5/PI3K/AKT, HR, NHEJ, and Bcl-2 [117]. Son et al. [100] demonstrated that quercetin was responsible for apoptosis in oral squamous cell carcinoma (YD10B and YD38 cells) via activating G1 cell cycle arrest, CDK inhibitor, cleavage of PARP, and p38 MAPK [100]. In the context of malignant melanoma, cancer quercetin induced apoptosis in B16 murine melanoma cells at the concentrations of 50 *μ*g/mL via decreasing the level of BCL-2 [101]. Furthermore, the apoptotic effect of quercetin was investigated in glioblastoma cancer cells where quercetin showed apoptotic cell death by increasing ROS, Bax, caspase-3 and caspase-9, PARP and decreasing Bcl-2, *β*-catenin, AKT, and NF-*κ*B pathway [90]. All these findings suggest that quercetin has a great ability to induce apoptosis in different cancer cells (Figure 2).

#### 2.7.1. Antiproliferative Effect

The development and progression of cancer cells increases when there is a disruption in the cell cycle checkpoints and multiple pathways that control the cell cycle progression [118, 119]. Numerous cells controlling pathways including the JAK-STAT, c-Myc, and Ras proto-oncoproteins are essential to control both cell proliferation and apoptosis under particular conditions where different growth factors are constricted [120]. Through the activation of different signal-transferring cascades by the mitogen-activated protein (MAP), kinase pathways stimulate different physiological activities including cell proliferation, differentiation, and apoptotic cell death [121]. The PI3K/AKT/mTOR system is the most significant signaling pathway, which can regulate cell growth, proliferation, and death [67]. Therefore, the modification of these pathways by different natural products can be used for cancer treatment.

A recent study conducted by Zhou and his team demonstrated the remarkable role of quercetin in the inhibition of cell proliferation in non-small-cell lung cancer cells (BEAS-2B, A549, and H1299) at 12.5, 50, and 200 *μ*M concentrations by stimulating the levels of p-CDK1 and halting the PI3K/AKT pathway which reduced the concentrations of HR, NHEJ, and DDR to knockdown cell proliferation by inducing apoptosis [117]. Another study carried out by Hisaka and his colleagues revealed the antiproliferative activity of quercetin in oral squamous cell carcinoma cells (YD10B and YD38) by inducing apoptosis and V-EGFP at 100 *μ*M dose [99]. In the oral squamous cell carcinoma cells (YD10B and YD38), quercetin could inhibit proliferation by activating PARP, p38, MAPK, CDK inhibitor, and apoptosis [100]. There is also a different study which has been investigated the antiproliferative effect of quercetin in B16 murine melanoma cells at 50 *μ*g/mL [101]. In T24 bladder cancer cells, quercetin could halt cell proliferation at 10−80 µM concentrations [26]. Quercetin could block cell proliferation in breast cancer cell lines via increasing the sensitivity of BC to PTX and reducing the EGFR and ERK levels [115]. According to a study carried out by Li et al. [98], quercetin could diminish cell proliferation in esophageal cancer cells by inhibiting the NF-*κ*B pathway [98]. A recent study conducted by Ding et al. [88] revealed that quercetin could also block cell proliferation in gastric cancer cells (GC cells, AGS, and GES-1) by inducing ferroptosis, p-Camk2/p-DRP1pathway and reducing SLC1A5, NRF2, xCT, and GPX4 expressions at the dose of 40 *μ*M [88]. In lung cancer cells, quercetin could inhibit cell progression [89]. Quercetin could induce antiproliferative activity in both hepatocellular carcinoma and intrahepatic cholangiocarcinoma cells by halting the NF-*κ*B pathway [102, 122]. These investigations suggested that quercetin has the unneglectable ability to block the proliferation of different cancer cells (Figure 2).

### 2.8. Inhibition of Invasion and Migration

The propagation of cancer from the primary tumor to distant sites is facilitated by two basic cellular mechanisms: invasion and migration [123]. In cancer cells, cellular invasion and migration become unregulated which consequently causes metastasis [124–126]. Different studies have demonstrated that the inhibition of invasion and migration of cancer cells is thought to be a significant therapeutic target [127, 128]. Different pathways and mechanisms could stimulate the inhibition of the invasion of cancer cells as revealed by different studies. Findings found that proteins involved in the processes of migration and invasion including matrix metalloproteinases (MMPs), along with disintegrin and metalloproteinases (ADAMs), and ADAM with thrombospondin motifs (ADAMTS), L1 cell adhesion molecule (L1CAM), nucleostemin, and nestin. SOX2 is also a key regulator of the process of invasion and migration of cancer cells [129, 130]. Different pathways including TNF-*α*/NF-*κ*B/Snail, ERK pathway, AKT pathway, TGF-*β*, Wnt, and Notch pathways [131–133].

Numerous studies have revealed that quercetin could trigger the inhibition of invasion and migration of different cancer cells including breast, esophageal, lung, and prostate cancer cells. A study carried out by Zhu and his coworkers found that quercetin could stimulate cell migration by inducing IL-2, IFN-*γ*, and IL-10 levels while reducing ASC, NLRP3, 5-HT, DA, and NE concentrations in breast cancer cells [29]. Similarly, in esophageal cancer cells (Eca109 and CLR-1730), quercetin could suppress cell migration and invasion by lowering the concentrations of VEGF-A and MMP-2 and MMP-9 at the dose of 10 *μ*g/mL [134]. Quercetin also blocked MAPK and NF-*κ*B pathways in esophageal cancer cells [98]. Quercetin could downregulate the levels of cytoplasmic HuR, *β*-catenin (HuR-dependent), and CD44 in TNBC breast cancer cells (MDA-MB-231 and MDA-MB-468) at 20–200 µM where the IC_50_ was 90 *μ*M and 98 *μ*M, respectively [135]. Additionally, quercetin could diminish cell migration by suppressing MGMT, Wnt3a, *β*-catenin, AKT, and NF-*κ*B in glioblastoma cancer cells [90]. Moreover, in hepatocellular carcinoma and intrahepatic cholangiocarcinoma cells, quercetin could halt the migration and invasion of cancer cells by increasing LC3 II/I and decreasing G-CSF, PD-L1, TNF-*α*, IL-6, and IL-17A and NF-*κ*B [102, 122]. So, quercetin could halt the invasion of cancer cells and give potential therapeutic (Figure 2).

#### 2.8.1. Inhibition of Angiogenesis

Cancer cells require oxygen and nutrients to survive and proliferate which can be supplied through the blood circulation system. These blood vessels are formed by a cellular process called angiogenesis which generates a new blood circulation system to provide nutrients and oxygen to the cancer cells [136, 137]. Numerous factors and pathways are involved in the process of angiogenesis. Different types of proteins like angiogenin, vascular endothelial growth factor (VEGF), basic fibroblast growth factor (bFGF), transforming growth factor (TGF)-*α*, TGF-*β*, tumor necrosis factor (TNF)-*α*, platelet-derived endothelial growth factor, granulocyte colony-stimulating factor, placental growth factor, IL-8, hepatocyte growth factor, and epidermal growth factor could stimulate angiogenesis. On the other hand, interferon, angiostatin, endostatin, platelet factor 4, thrombospondin, prolactin 16 kd fragment, and tissue inhibitors of metalloproteinase-1, metalloproteinase-2, and metalloproteinase-3 could suppress angiogenesis to prevent different types of cancer [138]. Therefore, targeting this angiogenesis stimulatory or inhibitory factors became the focus of therapeutic interventions. Different natural products could inhibit angiogenesis in cancer cells [139, 140].

An investigation carried out by Liu and his team members demonstrated that quercetin could suppress angiogenesis in esophageal cancer cells (Eca109 and CLR-1730) by diminishing the expression of VEGF-A and MMP-2 and MMP-9 at 10 *μ*g/mL concentrations [134]. Another study found that quercetin could significantly reduce angiogenesis to prevent cancer cells in the GBM xenograft mouse model [141]. These findings revealed the remarkable angiogenesis capability of quercetin in different cancer cells (Figure 2).

#### 2.8.2. Autophagy

Autophagy is a natural cellular process that breaks down and removes misfolded proteins and damaged organelles. It plays a role in responding to starvation, growth, cell death, and preventing tumor growth [142]. Autophagy can have multiple impacts on cancer, acting as either neutral, tumor-suppressive, or tumor-promoting depending on the specific circumstances and phases of cancer progression [143, 144]. A chain reaction of proteins regulates the mechanism of the autophagic process. Research studies have shown that the serine/threonine kinases, Beclin1, microtubule-associated protein light chain, p62, mTOR, AMPK, and autophagy-ATG genes are strongly preserved across different species and have a vital function in tightly controlling the autophagy process [145–147]. Activation of mTORC1 is crucial in phosphorylating autophagy-related protein (ATG) and subsequently suppressing autophagy [148]. Moreover, the suppression of the PI3K/AKT/mTOR signaling mechanism and the initiation of endoplasmic reticulum (ER) stress enhance the autophagy process [149]. In certain conditions, pharmacological autophagy modulation offers great promise as a novel cancer treatment, expanding the present repertoire.

Numerous studies have demonstrated that quercetin increases autophagy in cancer cells. Quercetin at concentrations of 50 *μ*M for 24 h in colorectal cancer cells (HepG2, Hep3B, MDA-MB-231, Atg7-WT, Atg7-KO MEF, and HCT116) resulted in the stimulation of autophagy [87]. Another study revealed that quercetin induced autophagy in AGS and MKN45 cells via increasing apoptosis and decreasing GSH, MDA, and ROS, Beclin1, and LC3B in gastric cancer [108]. Additionally, Guo et al. [27] showed that quercetin induced autophagy in A549 and H1299 cell lines, *in vitro* at different concentrations (12.5, 25, 50, and 100 *μ*M) for 24 h in lung cancer via upregulating the LC3-II, Beclin1, Atg5, Atg7, and Atg12, SIRT1 protein, and the pAMPK-AMPK as well as downregulating p62 [27]. In the context of hepatocellular carcinoma, Wu et al. [122] reported that quercetin enhanced autophagy in H22 and HepG2 cells, *in vitro* and *in vivo* by increasing LC3 II/I, and decreasing GM-CSF, G-CSF, PD-L1, p62, TNF-*α*, IL-6, IL-17A, and NF-*κ*B pathway [122] (Figure 2).

### 2.9. Anticancer Activity of Quercetin-Loaded Nanoformulations

Nanotechnology has completely transformed the field of cancer diagnosis and treatment. Nanoparticles within the size range of 1–100 nm have distinct characteristics that make them ideal for cancer treatment. These advantages include biocompatibility, lessened toxicity, higher stability, improved permeability, and absorption effect, as well as precision targeting capabilities [150, 151]. Nanotechnology-based transporters are highly effective for consecutive combination therapy because of their ability to encapsulate several cargos and deliver them precisely to cancer cells. Nanoformulations have demonstrated synergistic anticancer effects [152, 153]. Different studies suggested that natural product-based nanoformulations have more efficiency and eliminated side effects associated with monotherapy, including less aqueous solubility, poor bioavailability, multidrug resistance, and nonspecificity [154, 155].

**Figure 4:**
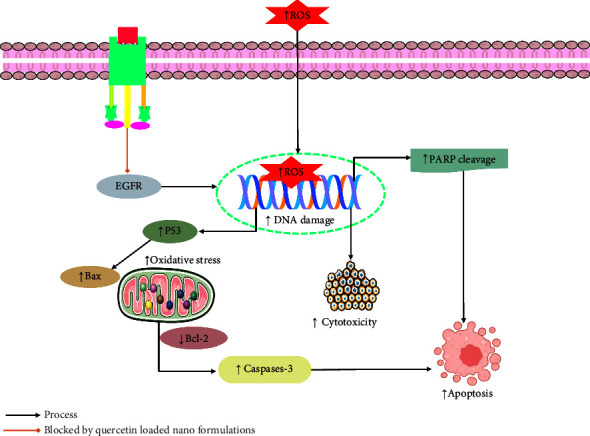
Mechanism of action of quercetin-loaded nanoparticles. EGRF: epidermal growth factor receptor; Bax: BcL2-associated X protein; p53: tumor protein p53; PARP: poly (ADP-ribose) polymerase; Bcl-2: B-cell lymphoma; ROS: reactive oxygen species.

In breast cancer cell lines, different quercetin-loaded nanoformulations showed higher anticancer efficacy compared to free quercetin therapy [155–157]. A recent study carried out by Sun and his colleagues demonstrated that the drug-carrying micelles (dHAD-QT), a combination of amphiphilic hyaluronic acid polymers (dHAD) with quercetin, showed strong cytotoxicity and apoptotic effect in breast cancer cell lines. dHAD-QT also diminished cell growth [155]. Another study conducted by Mohammed et al. [157] found that poly(d,l)-lactic-co-glycolic acid (PLGA)-encapsulated quercetin nanoparticles (Q-PLGA-NPs) could remarkably induce apoptosis, gene expression, cytotoxicity, Bax, and caspases-3 while reducing Bcl-2 and p53 expressions in breast cancer cells (MCF7 and CAL51cell) at different concentrations [157]. The findings of Liu et al. [156] also revealed the strong anticancer effects of quercetin (QC) coloaded and chondroitin sulfate (ChS)-coated mesoporous silica nanoparticles (MSNs) (MSNs-ChS@PQ) which could synergistically stimulate apoptotic cell death, G2/M phase arrest, and cytotoxicity in breast cancer cells at the dose of 1.5–45 *μ*g/mL [156]. In colorectal cancer cells (SW48), quercetin-loaded nanoliposomes showed greater anticancer activities than free quercetin (3–50 *μ*g/mL) by inducing cytotoxicity and apoptotic cell death and reducing *EGFR* gene expression [158]. A different study investigated the strong anticancer efficacy of quercetin-loaded chitosan in metastatic bone tumor cell (SH-SY5Y) at different concentrations (0.5, 1, 2, 4, and 8 *μ*g/m) compared to free quercetin treatment. At 2 *μ*g/mL dose, quercetin-loaded chitosan nanoparticles (NPs) reduced cell viability and significantly stimulated the expressions of 8-oxo-dG, cleaved caspase-3, Bax, cleaved PARP, oxidative stress, DNA damage, apoptotic cell death, and cytotoxicity (Figure 4) [159]. These investigations proved the greater efficiency of nanoformulated quercetin in contrast to free quercetin treatment in different cancer cells as it enhanced bioavailability and solubility of quercetin (Table 2). Although quercetin nanoformulations have been extensively studied for their anticancer properties in laboratory and animal experiments, there are still several obstacles that hinder their practical application in medical clinical applications, including high cost, safety concerns, and potential side effects. Moreover, liposomes exhibit certain drawbacks as a carrier for drugs, such as limited capacity for drug loading, reduced stability, high costs, and active targeting [160, 161]. The mechanism of action of quercetin-loaded nanoparticles is depicted in Figure 4.

### 2.10. Anticancer Activity of Quercetin in Combination with Other Molecules

The process of combining two or more therapeutic compounds in order to increase the efficacy of a drug in contrast to the single therapy method is known as combination therapy. In combination therapy, each compound targets different key signaling pathways in a synergistic way which leads to reduce the required concentrations for individual drugs and consequently minimizes the cost of therapeutic treatment along with increasing the efficiency of the drug compared to monotherapy [162, 163]. It can also decrease the drug resistance possibility and, at the same time, induce different anticancer activities, including reducing cell growth, blocking cell cycle arrest, halting cell proliferation, and inducing apoptosis [164].

**Figure 5:**
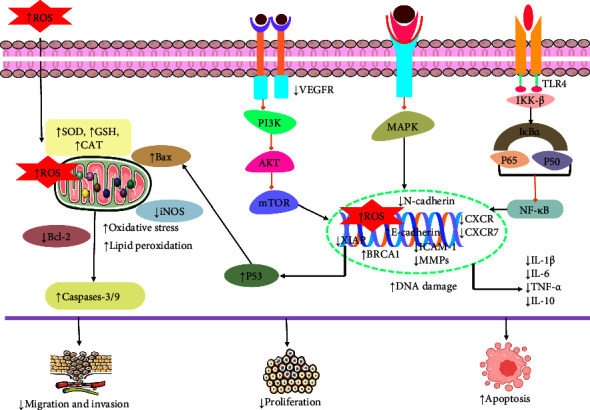
Possible anticancer mechanism of quercetin in combination therapy. XIAP: X-linked inhibitor of apoptosis protein; N-cadherin: neural cadherin (protein); p38: the serine/threonine kinase p38; iNOS: inducible nitric oxide synthase; ICAM-1: intercellular adhesion molecule 1; MMP-2: matrix metalloproteinase-2; CXCR: chemokine receptors; CAT: catalase; SOD: superoxide dismutase; PCNA: proliferating cell nuclear antigen; VEGF: vascular endothelial growth factor; IKK*β*: inhibitor of nuclear factor kappa-B kinase subunit beta; xIAP: X-linked inhibitor of apoptosis protein; AKT: protein kinase B; IL-1*β*: cytokine interleukin-1*β*; ROS: reactive oxygen species; Bax: BcL2-associated X protein; Bcl-2: B-cell lymphoma; p53; MAPK: mitogen-activated protein kinases; p65: transcription factor p65; PI3K: phosphoinositide 3-kinase; IFN-*γ*: interferon-gamma; E-cadherin: epithelial cadherin; NF-*κ*B: nuclear factor-*κ*B.

A current investigation carried out by Tanomrat et al. [165] demonstrated that quercetin and N-acetylcysteine (NAC) demonstrated strong anticancer activity in colorectal cancer cells (HT-29 and HCT-116) by increasing ROS levels and decreasing iNOS, ICAM-1, MMP-2, cell progression, migration, and invasion at the dose of 0.5 *μ*g/ml of quercetin combined with 0.125 and 0.25 mM NAC in HT-29 cells; additionally, 10 *μ*g/ml of quercetin combined with 2.5 and 5 mM NAC in HCT-116 cells [165]. Different studies revealed the efficacy of combination therapy in breast cancer cells compared to monotherapy [166–168]. Quercetin and curcumin combinedly induced BRCA1, and histone acetylation and reduced cell survival and migration at 50 *μ*M dose in triple-negative breast cancer cells. These findings suggested that the combination of quercetin and curcumin demonstrated strong anticancer activities in breast cancer cells [167]. A study conducted by Hanikoglu and his team revealed the great anticancer activity of the combination of quercetin with curcumin and somatostatin could reduce p-S6-Ribosomal (Ser235/236), omega-3 acids, AKT 1 and p-AKT 1, EGFR, and MAPK in breast cancer cell lines (MCF-7 and MDA-MB231) [169]. Another study carried out by Rhman et al. [168] demonstrated the strong anticancer efficacy of quercetin and naringenin (CoQN) in breast cancer cells. They could stimulate oxidative stress, apoptosis, cytotoxicity, caspase 3/7, miR-1275, and lipid peroxidation. At the same time, they could diminish cell viability, Bcl-2, cell proliferation, MMP, and miR-27a-3p [166, 168]. A combination of quercetin and fisetin could also induce anticancer activities synergistically in breast cancer cells via upregulating apoptotic pathways and suppressing cell proliferation, migration, colony formation, and MMP [170]. In prostate cancer cells (PC3 and DU145), quercetin and vitamin C increase the anticancer activities synergistically through reducing CXCR and CXCR7; *α*4, *α*5, and *β*1 integrin subunits; VEGF; Ki-67; cell proliferation at the dose of quercetin (75 µM); and vitamin C (100 *μ*M) [171]. Combination of quercetin and naringenin could stimulate cell cycle arrest at G0/G1 and S phases and apoptotic cell death, ROS, and cytotoxicity in hepatocellular carcinoma cell (HepG2). They also diminished cell migration and invasion simultaneously [172]. A different study carried out by Roy and his colleagues investigated that quercetin and ruthenium could induce p53, CAT, SOD, glutathione levels, Bax, and apoptotic cell death in colon cancer cells (HT-29). They also reduced the expression of VEGF and mTOR, Bcl-2, PCNA, and cell proliferation at different concentrations (10, 15, 20, 30, and 40 mM) [173]. In thyroid cancer cell, quercetin and sorafenib could remarkably increase E-cadherin and decrease cell proliferation, cell adhesion and migration properties, N-cadherin, and cell growth [174]. Besides, in human colorectal adenocarcinoma cells, a combination of quercetin, luteolin, and 5-FU could stimulate apoptosis and unneglectable knockdown VEGF, cell growth, Bcl-2, mTOR protein, and *AKT* gene at 50–1000 mg/ml concentrations [175]. The combination of quercetin and chrysin could downregulate the levels of TLR4/NF-*κ*B, invasion and migration, p65, cytokines, IL-1*β*, IL-6, TNF-*α* and IL-10, TLR4, Myd88, phosphorylation of IKK*β*, I*κ*B, and MMP-9 in lung cancer cells (H1975 and A549) at 2 or 5 *μ*M [114]. A study conducted by Li and his team revealed that quercetin and cisplatin increased apoptotic cell death and caspase-8 and caspase-9 while decreasing NF-*κ*B, AKT, IKK*β*, xIAP, and cell growth in oral cancer cells (Tca-8113 and SCC-15) at 3−6 mg/kg doses (Figure 5) [176]. According to a study conducted by Karimian et al. [177], quercetin and thymoquinone significantly enhanced the anticancer activities in breast cancer cells (MCF-7), lung cancer cells (A549), and prostate cancer cells (PC3) through stimulating DNA damage markers, H2AX, 8-OH-dG, DNA damage, and cytotoxicity. Additionally, the study demonstrated that quercetin and thymoquinone could suppress the levels of DNA repair mediators, RAD51, Ku70, XRCC1, cell proliferation, and P53 [177]. All these findings demonstrated the strong synergetic anticancer activities of different combination therapies over monotherapy in different cancer cells at low concentrations of individual drugs (Table 3). The mechanism of action of quercetin in combination therapy is depicted in Figure 5.

### 2.11. Role on Immunotherapy

The immune system plays a significant role in immune defense, immune surveillance, and immune homeostasis. The development of cancer is an outcome of reduced immunity because when the immune response is decreased, aging-damaged mutant cells or invasive pathogens cannot be eradicated in a timely manner. Therefore, enhancing immune response is very important to prevent cancer cell growth [178, 179]. The field of cancer immunotherapy (neoadjuvant and adjuvant immunotherapies) has been significantly revolutionized by immune checkpoint blockade therapy [180, 181]. Different signaling proteins such as TNF-*α*, PD-L1, IL-6, NRF2IL-2, and IFN-*γ* as well as immune cells (CD4+ and CD8+ T) regulate the immune response which can be the target for anticancer drugs to prevent cancer cell proliferation and cell growth [29, 182, 183]. By understanding the mechanism of tumor immune response and its evasion by tumors, the field of immunotherapy can manipulate this interaction and elucidate the therapeutic function of immunity in cancer. This improved understanding of immunotherapy and the mechanisms underlying immunity in cancer has opened a new door to developing a wide range of therapeutic agents for treating a variety of cancers [184]. Quercetin not only has anti-inflammatory, antitumor, antiplatelet aggregation, endothelial cell protection, and antioxidant effects but also has a regulatory effect on immune cells to control immune responses [179].

Different studies revealed that quercetin remarkably showed an immune response to prevent tumor growth in different cancer cells. In LPS-treated 4T1 cells, quercetin significantly stimulated immune response and upregulated the levels of IL-2, IFN-*γ*, and IL-10 [29]. NRF2 played an important role in inducing oxidative stress and immune response in gastric cancer cells at 40 *μ*M [88]. A study conducted by Wu and his team revealed that quercetin notably reduced TNF-*α*, IL-6, PD-L1, and IL-17A to enhance the immune response to resist cancer cell proliferation in hepatocellular carcinoma cells (H22 and HepG2) [185] (Table 4). Another study conducted by Qiu and his team found the immune therapeutic activity of quercetin in breast cancer cells (MCF-10A, MCF-10AT, MCF-7, and MDA-MB-231). Quercetin stimulated apoptosis and differentiation of *γδ* T cells into the V*δ*2 T-cell subpopulation. It also induced the level of IFN*γ*-R, p-JAK2, and p-STAT1 and decreased PD-L1 level [179]. On the other hand, nanoformulated quercetin displayed a synergistic immune response compared to free quercetin treatment. According to a study of Sun and his coworkers demonstrated that a cyclodextrin-based nanoformulation of ginsenoside Rg3 and quercetin (CD-PEG-FA.Rg3.QTN) and found that CD-PEG-FA.Rg3.QTN significantly induced immunogenic cell death (ICD) by stimulating effector T cells and inhibiting CD4+ or CD8+ T cells in colorectal cancer cells (CT26 and HCT116). CD-PEG-FA.Rg3.QTN also reduced immunosuppressive tumor microenvironment (TME) and increased blood circulation and antiproliferative effects with IC50 of 32 *μ*mol/L and 30 *μ*mol/L, respectively [181]. The tumor microenvironment could suppress the immune response [186]. In microsatellite-stable colorectal cancer, the micellar delivery of quercetin and alantolactone (QA-M) potentially enhanced ICD, toxicity, immune response, and memory tumor surveillance as well as diminished tumor growth and immunosuppressive TME [187] (Table 5). These findings suggested that quercetin has the potential ability to treat cancer through immunotherapy.

### 2.12. Clinical Evidence

Polyphenolic substances called flavonoids have the potential to be used as therapeutics. Numerous investigations have demonstrated the stronger anticancer potential of these substances. Quercetin is one of the most significant flavonoids among them found in the human diet [188]. Different studies revealed that quercetin has shown remarkable anticancer and anti-inflammatory activities, mainly due to the potential for antioxidants [189–191].

Quercetin lacks sufficient clinical evidence to support it as a significant anticancer drug. However, there is a few numbers of clinical studies of quercetin in cancer and other diseases. A clinical study carried out by Henning and his coworkers found the great anticancer activity of quercetin in prostate cancer cells. They used 31 male subjects to investigate, and they combined 1 gram of green tea extract (GTE) with 800 mg of quercetin. They revealed that quercetin could induce bioavailability, epigallocatechin (EGC) levels in urine, plasma epigallocatechin, and the accumulation rate in plasma, urine, and prostate tissue. Quercetin also reduced methylation activity and liver toxicity [192]. Another study demonstrated that quercetin could decrease lymphocyte tyrosine kinase activity and the serum alpha-fetoprotein where t(1/2) alpha of 6 min and median t(1/2) beta of 43 min in 11 patients with ovarian cancer. Quercetin was infusion at escalating doses initially at 3-week intervals. The first dose level was 60 mg/m2, and the 10th dose level was 1700 mg/m^2^ [193]. In the United Kingdom, Morrow et al. [194] conducted a clinical study that revealed the strong antitumor activity of quercetin in 4 healthy males which could decrease the level of TIMP-1 in the cell at the dose of 30 mg per day for 14 days. But it has some side effects as it could cause aggressive disease and poor prognosis in patients with certain malignancies [194]. Apart from cancer treatment, quercetin is also investigated to treat different diseases. The combination of quercetin and ascorbic acid (97 mg quercetin and 16 mg ascorbic acid a day) could upregulate plasma concentrations of quercetin and ascorbic acid and Trolox equivalent antioxidant capacity (TEAC) and oxidative DNA damage in 114 females and 54 males (aged 18–45 years) [195]. Besides, in polycystic ovary syndrome, quercetin could decrease the number and size of ileal and rectal adenomas in 72 women at concentrations of 500 mg for 40 days [196]. Cruz-Correa and his team revealed that quercetin and curcumin could suppress the number and size of ileal and rectal adenomas in five FAP patients with prior colectomy (4 with retained rectum and 1 with an ileal anal pouch) in Weston, Florida, USA. However, there were some limitations like it caused nausea and the taste is sour [197] (Table 6). Though a significant number of *in vivo* and *in vitro* studies demonstrated the anticancer activities of quercetin in different cancer cells, more clinical studies should be conducted to support quercetin as an anticancer drug.

#### 2.12.1. Toxicological Profile

It has been demonstrated that numerous drugs can undergo conversion into different metabolites within the body, which elicit both therapeutic and toxicological effects [198, 199]. One of the major challenges in drug discovery and development is the toxicity of natural products and isolated chemicals. So, an in-depth analysis is always necessary in search of safer natural medications [200]. Toxicological biomarkers of natural products can be found and evaluated using metabolomics-based toxicology, which helps to prevent adverse drug reactions and guide clinical medication. Numerous metabolomic studies have been conducted over the past few decades to evaluate toxicity, identify toxicological biomarkers, and investigate the primary pathways of nephrotoxicity, hepatotoxicity, cardiotoxicity, and central nervous system toxicity stimulated by different natural compounds [35, 201]. The process of toxicity testing is a significant step in the drug manufacturing process. The toxicity of an investigational substance is highly specific based on the species, organ, and dose which are investigated through different preclinical toxicity studies. The toxicity test can be conducted in different ways, including by studying accidental exposures to a compound, *in vitro* studies using cells and animal studies [202]. The median lethal dosage (LD50) is used to determine the short-term toxic effect of the compound [203].

Different investigations carried out by researchers demonstrated that quercetin had no adverse effect on the survival of the animal and did not show any toxicity. A toxicological study revealed no toxicity of quercetin which was conducted in male and female F344/N rats at the dose of 40–1900 mg/kg/day for 2 years. However, a high dose could reduce the body weight of the mice [204]. A combination study of 4-chloromercuribenzoic (pCMB) acid and quercetin showed no remarkable changes in physiological, behavioral, and serum biochemical properties in Swiss albino mice in the oral administration where the LC50 was 91.57 ± 0.35 mg/L and 448.45 ± 0.46 mg/L, respectively, though they showed a minor toxicity in high dose [205]. An *in vivo* and *in vitro* study carried out by Han and his team members found that quercetin could suppress LPS-induced microglial toxicity and neurodegeneration in PD mouse models. It also halted neuronal injury through the blocking of mtROS-mediated NLRP3 inflammasome activation in microglia by mitophagy stimulations [206]. Another subchronic toxicity demonstrated the safety of quercetin in male and female CD2F1 mice at several concentrations (62, 125, and 250 mg/kg of diet) for 98 days [207]. Quercetin could prevent Cd-induced toxicity via moderating energy and lipid metabolism, stimulating the antioxidant protective mechanism, and protecting liver and kidney function in sixty male Sprague Dawley rats at different concentrations (10–50 mg/kg bw/day) [156].

These findings demonstrated that quercetin exerts significant anticancer activity while it has no toxic effect at low doses. Therefore, further investigation is required to investigate the potentially toxic effects of quercetin following chronic ingestion.

## 3. Conclusion and Future Perspectives

In spite of significant advancements in the treatment approach that have been established in recent years, cancer still remains a most fearsome disease which causes a significant number of deaths around the world each year. Though chemotherapy was thought to be the most useful treatment for cancer, it has numerous side effects on human health. In recent years, several natural products have been used in the drug discovery process for cancer treatment as they have shown strong anticancer activities. Quercetin, a flavonoid compound, is used in the medications of different diseases, including cancer, inflammation, and neurological disorders. This study has unveiled the remarkable anticancer activities of quercetin in different cells, including bladder, breast, colon, colorectal, esophageal, gastric, lung, prostate, liver, and oral squamous cell carcinoma (OSCC) cells. Quercetin showed anticancer properties like apoptosis, cell cycle arrest, oxidative stress, inhibition of angiogenesis, inhibition of cell proliferation and migration, ferroptosis, and cytotoxicity through different mechanisms and pathways including suppression of EGFR and ERK, PI3K/AKT/mTORC1 pathway, regulating the JAK/STAT signaling system, diminishing the MAPK signaling pathway, modulation of the MMP signaling pathway, suppression of NF-*κ*B pathways, upregulation of p-Camk2/p-DRP1, and SIRT5/PI3K/AKT pathway. Different studies investigated that quercetin significantly accumulated in the lungs, liver, kidneys, and small intestines at high concentrations and in the brain, heart, and spleen at low concentrations while excreted through the renal, fecal, and respiratory systems. The oral bioavailability of quercetin is very low, and it shows low water solubility as well as a short biological half-life. So, it requires alternative ways of administration. In this case, combination with other molecules or nanoformulation treatment could give potential effects of quercetin to overcome these limitations. This study also described that quercetin showed synergistic effects in both combination therapy and nanotherapy which will be helpful in the future to develop innovative therapeutics. The updated nanosystems discussed in the literature demonstrated promising methods for effectively encapsulating quercetin and releasing it in a controlled way. Nanotechnology provides an opportunity to produce nanoparticles loaded with phytochemicals, which can be used to prevent and treat cancer. In addition, several techniques for manipulating nanoparticles have enabled the precise delivery of quercetin to specific tissues in tumor microenvironments, as well as enhancing its ability to cross the blood-brain barrier and penetrate within skin layers. But it has different side effects and it is a costly process. So, future research should be conducted to develop more effective nanoformulation of quercetin with low cost and minimal side effects. Addressing the challenge of quercetin′s poor bioavailability, different studies revealed that complexation with other molecules or nanoparticles notably enhanced the bioavailability of quercetin. These findings indicated that future research on the discovery of novel pharmaceutics by combination or nanoformulation process indicating a wide spectrum of potential medical applications. These recommended future investigations will determine the potential of quercetin to be used as a potential drug in the field of oncology. This study also found that quercetin demonstrated safety in the animal clinical trial and no toxicity was observed. However, there is a lack of clinical studies to support quercetin as an effective drug in cancer treatment. So, in conclusion, this study suggested that more clinical trials should be conducted to find out the strong anticancer activities of quercetin and to support quercetin as a significant anticancer drug.

## Figures and Tables

**Table 1:** Botanical sources of quercetin.

Plant	Part (s)	References
*Alchornea glandulosa*	Leaves	[40]
*Allium cepa* L.	—	[41]
*Bergia ammannioides*	Aerial	[42]
*Fraxinus angustifolia*	Leaf and bark	[43]
*Leonurus sibiricus*	Whole plant	[44]
*Melilotus officinalis*	—	[45]
*Sambucus ebulus* L.	Leaves	[46]
*Rubus niveus*	Roots	[47]
*Tephrosia purpurea*	Aerial	[48]
*Thea sinensis*	—	[49]
*Glycyrrhiza glabra*	—	[49]
*Hypericum perforatum*	—	[49]
*Ginkgo biloba*	—	[49]
*Styphnolobium japonicum* L. *Schott*	Flower and flower bud	[50]
*Lannea coromandelica*	Stem bark	[51]
*Solidago canadensis* L.	Aerial part	[52]
*Solidago gigantea Ait.*	Aerial part	[52]
*Pseudocydonia sinensis* C. K. *Schneid*	Fruit	[53]
*Hancornia speciosa*	Leave	[54]
*Waltheria indica* L.	—	[55]
*Quillaja saponaria Mol.*	—	[56]
*Physalis lagascae*	Whole plant	[57]
*Adiantum capillus-veneris*	Leave	[58]
*Clitoria ternatea*	Flower	[58]
*Holarrhena antidysenterica*	Seed	[59]
*Helichrysum armenium* subsp. *armenium*	Aerial part	[60]

**Table 2:** Anticancer activity of different quercetin-loaded nanoformulations

Types of cancer	Name of the compound	Experimental model	Tested concentration	Efficacy, IC_50_ (exposure time)	Anticancer effects and mechanisms	Ref.
Breast cancer	dHAD-QT micelles	BC cells, *in vivo*	—	—	↑Cytotoxicity and apoptotic cell death, ↓cell growth	[155]
	Poly(d,l)-lactic-co-glycolic acid (PLGA)-encapsulated quercetin nanoparticles (Q-PLGA-NPs)	MCF7 and CAL51cell lines, *in vivo and in vivo*		—	↑Apoptosis, gene expression, and cytotoxicity, Bax, caspase-3, ↓Bcl-2, p53	[157]
	Quercetin (QC) coloaded and chondroitin sulfate (ChS)-coated mesoporous silica nanoparticles (MSNs) (MSNs-ChS@PQ)	MCF-7/ADR cells, *in vivo and in vitro*	1.5−45 *μ*g/mL	—	↑Apoptotic cell death, G2/M phase arrest, cytotoxicity, ↓P-gp	[156]

Colorectal cancer	Quercetin-loaded nanoliposomes	SW48 cell*, in vitro*	3–50 *μ*g/mL	10.65 *μ*g/mL	↑Cytotoxicity, apoptotic cell death, ↓*EGFR* gene expression	[158]

Metastatic bone tumor	Quercetin-loaded chitosan nanoparticles (NPs)	SH-SY5Y cell, *in vitro*	0.5, 1, 2, 4, and 8 *μ*g/m	2 *μ*g/mL	↑8-oxo-dG, cleaved caspase-3, Bax, cleaved PARP, and total oxidant, oxidative stress, DNA damage, apoptotic cell death, cytotoxicity, ↓cell viability	[159]

Arrows (↑ and ↓) show an increase and decrease in the obtained variables, respectively. Bax: BcL2-associated X protein; P-gp: P-glycoprotein 1; EGRF: epidermal growth factor receptor; RAD51: RAD51 homolog 1; P53: tumor suppressor gene.

**Table 3:** Anticancer activity of quercetin with other molecules in combination therapy.

Types of cancer	Name of the compound	Experimental model	Tested concentration	Efficacy, IC_50_ (exposure time)	Anticancer effects and mechanisms	Ref.
Colorectal cancer	Combination of quercetin and N-acetylcysteine (NAC)	HT-29 and HCT-116 cells	The HT-29 cells were treated with 0.5 *μ*g/ml of quercetin combined with 0.125- and 0.25 mM NAC. The HCT-116 cells were treated with 10 *μ*g/ml of quercetin combined with 2.5 and 5 mM NAC	—	↑ROS, ↓cell progression, migration and invasion, iNOS, ICAM-1, and MMP-2	[165]

Triple-negative breast cancer (TNBC)	Combination of quercetin and curcumin	MDA-MB-231, MDA-MB-468 cells , induced by BRCA1 siRNA-induced, *in vitro*	50 *μ*M	—	↑BRCA1, histone acetylation, ↓cell survival and migration	[167]

Breast cancer	Combination of quercetin with curcumin and somatostatin	MCF-7 and MDA-MB231 cells, *in vitro*	MCF7 cells with the EC50 concentrations of somatostatin combination of quercetin (38.58 *μ*M), curcumin (29.65 *μ*M) and quercetin (73.63 *μ*M) for 24 h. For MDA-MB-231 cells, EC50 concentrations of somatostatin was 96.75 *μ*M, curcumin was 10.46 *μ*M, and quercetin was 92.98 *μ*M	—	↓p-S6-Ribozomal (Ser235/236),omega-3 acids, AKT 1 and p- AKT 1, EGFR and MAPK	[169]
	Combination of quercetin with naringenin (CoQN)	MCF-7 BC cells, *in vitro*	50 *μ*g/mL of quercetin with naringenin	44.31 *μ*g/mL	↑Oxidative stress and apoptosis, cytotoxicity, caspase 3/7, lipid peroxidation, ↓cell viability, Bcl-2 ,cell proliferation, MMP	[168]
	Combination of quercetin and naringenin (CoQN)	MCF7 and MDA-MB-231 breast cancer cell lines, *in vitro*	—	—	↑Apoptotic cell death, miR-1275, ↓cell growth, migration, miR-27a-3p.	[166]
	Combination of quercetin and fisetin	MCF7, MDA-MB-231, BT549, T47D, and 4T1, *in vivo and in vitro*	—	—	↑Apoptotic pathways, ↓cell proliferation, migration, and colony formation, MMP	[170]

Prostate cancer	Combination of quercetin and vitamin C	PC3 and DU145 cell lines, *in vitro*	quercetin (75 *μ*M) and vitamin C (100 *μ*M)	—	↓ CXCR and CXCR7, *α*4, *α*5 and *β*1 integrin subunits, VEGF, Ki-67, proliferation	[171]

Hepatocellular carcinoma	Combination of quercetin and naringenin	HepG2 cell, *in vitro*	naringenin and quercetin 200 and 100 *μ*M respectively	—	↑Cell cycle arrest at G0/G1 and S phases and apoptotic cell death, ROS, cytotoxicity, ↓ cell migration, invasion	[172]

Colon cancer	Combination of quercetin and ruthenium	HT-29 cells, *in vitro and in vivo*	10, 15, 20, 30, 40 mM	—	↑p53, CAT, SOD and glutathione levels, Bax, apoptotic cell death, ↓VEGF and mTOR, Bcl-2, cell proliferation, PCNA	[173]

Thyroid cancer cells	Combination of quercetin and sorafenib	K1 and BCPAP cells, *in vivo.*	sorafenib 0.1 *μ*M, quercetin 25 *μ*M	—	↑E-cadherin, ↓cell proliferation, cell adhesion and migration properties, N-cadherin, cell growth	[174]

Human colorectal adenocarcinoma	Combination of quercetin and luteolin and 5-FU	HT 29 cells, *in vitro*	(50–1000 mg/ml) for 24 h	—	↑Apoptotic cell death, p53, Bax, p38 MAPK, and PTEN, ↓VEGF, growth , Bcl-2, mTOR, and AKT gene	[175]

Lung cancer	Combination of quercetin and chrysin	H1975 and A549cells, *in vitro*	2 or 5 *μ*M	—	↓TLR4/ NF-*κ*B, invasion and migration, p65, cytokines, IL-1*β*, IL-6, TNF-*α* and IL-10, TLR4 ,Myd88, phosphorylation of IKK*β* and I*κ*B, MMP-9	[114]

Oral cancer	Combination of quercetin and cisplatin	Tca-8113 and SCC-15 cells, *in vitro and in vivo*	quercetin and cisplatin (3−6 mg/kg), cisplatin (2 mg/kg) , quercetin (50 mg/kg)	—	↑Apoptotic cell death,caspase-8 and -9, ↓NF-*κ*B, AKT, IKK*β*, xIAP, growth	[176]

Breast cancer	Combination of quercetin and thymoquinone	MCF-7 cells, *in vitro*	—	50 *μ*M	↑DNA damage markers, H2AX, and 8-OH-dG, DNA damage, cytotoxicity, ↓DNA repair mediators, RAD51, Ku70, XRCC1, cell proliferation, P53	[177]
Lung cancer		A549 cells, *in vitro*	—	200 *μ*M		
Prostate cancer		PC3 cells, *in vitro*	—	400 *μ*M		

Arrows (↑ and ↓) show an increase and decrease in the obtained variables, respectively. XIAP: X-linked inhibitor of apoptosis protein; N-cadherin: neural cadherin (protein); p38: the serine/threonine kinase p38; iNOS: inducible nitric oxide synthase; ICAM-1: intercellular adhesion molecule 1; MMP-2: matrix metalloproteinase-2; CXCR: chemokine receptors; CAT: catalase; SOD: superoxide dismutase; PCNA: proliferating cell nuclear antigen; VEGF: vascular endothelial growth factor; IKK*β*: inhibitor of nuclear factor kappa-B kinase subunit beta; xIAP: X-linked inhibitor of apoptosis protein; AKT: protein kinase B; IL-1*β*: cytokine interleukin-1*β*; ROS: reactive oxygen species.

**Table 4:** Anticancer activity of quercetin against different cancers

Cancer type	Test system	Dose/concentration (R/A)	IC50	Results/possible mechanism	References
Bladder cancer	T24 bladder cancer cells, MTT assay and trypan blue exclusion test, *in vitro*	10−80 *μ*M	—	↑Apoptotic and senescence effect, cell death, cell cycle arrest, cytotoxicity, ↓proliferation, cell body, cytoplasmic retraction, and membrane condensation	[26]

Breast cancer	LPS-treated 4T1 cells, *in vivo and in vitro*	—	—	↑Immune response, IL-2, IFN-*γ*, and IL-10, ↓PYD ,ASC, NLRP3, and caspase-1, 5-HT, DA, and NE	[29]
	MCF-7 cell, *in vitro*	100 *μ*M	23.1 *μ*M	↑Cell cycle arrest at S phase, apoptosis, cytotoxicity, ROS, oxidative stress	[86]
	BC cell, *in vitro*	—	—	↑Apoptotic cell death, sensitivity of BC to PTX, ↓cell proliferation, EGFR, ERK	[115]
	MCF-7 and MDA-231 cells, *in vitro*	0.1, 1, and 10 *μ*M	—	↑Ferroptosis, iron, MDA, and carbonyl protein, LAMP-1, TFEB, malondialdehyde (MDA), carbonylation protein (CFP), ↓cell viability	[107]

C26 tumor (mice)	C26 cells*, in vitro in vivo*	4 mg/mL	—	↑Antioxidant, protein expression of mitochondrial complexes V, III, and II, cytochrome c expression, MFN1, BNIP3, ↓FIS1, TFAM protein	[208]

Colon cancer	*In vitro and in vivo* (n =15)	10 mg/kg		↑Caspase-3, apoptotic cell death, ↓beta-catenin, and Bcl-2 proteins	[33]
	HT-29 cells exposed to BPA, *in vitro and in silico*	—	160.63 *μ*M	↑Antioxidant, cell cycle arrest in the G0/G1, *ESR2* and *GPR30* genes, apoptosis, cell death, cytotoxicity, ↓cell viability	[32]
	HCT 116, COLO 320, and COLO 205 cells, *in vitro*	—	80 *μ*M in L132 cells and 120 *μ*M in all the colon cancer cell lines	↑Apoptosis, sirtuin-6 and klotho, cell cycle arrest, ↓cell proliferation, cell growth, proteasome 20S	[31]
	Colo-320 and Colo-741 cell lines, MTT assay, *in vitro*	25 *μ*g/ml quercetin for 48 hours	-	↑Apoptotic cell death , p16, lamin B1 and cyclin B1, senescence, cytotoxicity, Bax, cleaved caspase-3, ↓cell growth, Bcl-2, cell viability	[116]

Colorectal cancer cells	HepG2 cells, Hep3B, MDA-MB-231, Atg7-WT and Atg7-KO MEF cells, HCT116 cells, MTT assays*, in vitro*	50 *μ*M for 24 h	—	↑Cell death, autophagy, lysosomal activation, nuclear translocation of EB and transcriptional activation of lysosomal genes, cytotoxicity, cell death, TFEB, ferroptosis, ferritin degradation, free iron release, ROS, lipid peroxidation, Bid, cleavage of PARP and caspase-9, ↓mTOR, p53 , PI3K/AKT-mTORC1 pathway, MMP	[87]

Esophageal cancer	Eca109, CLR-1730 cells*, in vitro*	10 *μ*g/mL	—	↓Cell migration, invasion, angiogenesis, VEGF-A, MMP-2, and -9	[134]
	Esophageal carcinoma cells*, in vitro*		—	↑Cell cycle arrest, ↓cell proliferation, invasion, and clonal formation, MAPK, NF-*κ*B	[98]

Gastric cancer	GC cells, AGS cells, GES-1 cells*, in vitro and in vivo*	40 *μ*M	In AGS cells 38.78 *μ*M	↑Ferroptosis, p-Camk2/p-DRP1, cytotoxicity, ROS levels, ↓SLC1A5, NRF2, xCT, GPX4, cell progression	[88]
	AGS and MKN45 cells, *in vitro* BALB/c mice, *in vivo*			↑Ferroptosis, autophagy, and apoptosis, ↓cell viability and tumor volume, GSH, MDA, ROS, Beclin1, and LC3B	[108]

Lung cancer	A549 and H1299 cell lines, *in vitro*	12.5, 25, 50, and 100 *μ*M of quercetin for 24 hours	—	↑Apoptotic effect, autophagy, LC3-II, Beclin1, Atg5, Atg7, and Atg12 , SIRT1 protein and the pAMPK-AMPK, autophagy, cleaved caspase-3 protein, Bax, cytotoxicity, ↓cell viability, p62, Bcl-2	[27]
	Lung cancer cells, *in vitro*	—	—	↑ROS, caspase -2 and-3, DNA damage, S-phase cell cycle arrest, ATM, cell death, ↓cell proliferation	[89]

Prostate cancer	LNCaP, DU-145 with mutated p53 , and PC-3 cells, *in vitro and in vivo*	40 *μ*M	—	↑Apoptotic cell death, Raf, MEK, ROS, Bax, ↓cell viability, AKT pathway, NF-*κ*B pathways, Bcl-2	[28]

TNBC breast cancer	TNBC Cells (MDA-MB-231 and MDA-MB-468), *in vitro*	20–200 *μ*M	90 *μ*M and 98vM	↑Cytotoxicity, ↓cytoplasmic HuR, adhesion and migration, *β*-catenin (HuR-dependent) and CD44	[135]

Glioblastoma	GBM xenograft mouse model*, in vivo*	—	—	↑Apoptosis, ↓cell proliferation, cell growth, glycolytic metabolism, angiogenesis	[141]

Breast cancer, hepatocellular carcinoma, cervical carcinoma, and human ovarian carcinoma	MCF-7, SMMC-7721, HeLa, SKOV3 cell lines, *in vitro*	—	—	↑Apoptosis, G0/G1 phase and G2/M phase arrest, Bax, ↓cell proliferation, Bcl-2	[39]

Non-small cell lung cancer (NSCLC)	BEAS-2B, A549 and H1299 cells, *in vitro*	(12.5, 50, and 200 *μ*M) of quercetin for 24 h	—	↑Apoptotic cell death , caspase-3, Bax, DNA damage, p-CDK1, ferroptosis, ↓SIRT5/PI3K/AKT, HR, NHEJ, proliferation, Bcl-2, DDR	[117]

Liver cancer	KIM1, KYN-2, KYN-3, HAK-1B, HAK-2, HAK-5, HAK-6, KMCH-1, KMCH-2 cells, *in vitro*	100 *μ*M	—	↑Cell cycle arrest, apoptosis, V-EGFP, ↓cell proliferation	[99]

Oral squamous cell carcinoma (OSCC)	YD10B and YD38 cells*, in vitro*	—	—	↑Cytotoxic, G1 cell cycle arrest, CDK inhibitor, cleavage of PARP, p38 MAPK, apoptotic cell death, ↓cell Proliferation	[100]
	OSCC cell lines, *in vitro*	—	—	↑Ferroptosis, mTOR/S6KP70, lipid peroxidation, ↑tumor growth, GSH, SLC7A11	[106]

Malignant melanoma	B16 murine melanoma cells, *in vitro*	50 *μ*g/mL	—	↑Cell cycle arrest in the S and G2/M stages, apoptotic cell death, ↓cell viability, proliferation, BCL-2	[101]

Glioblastoma cancer	T98g cells, *in vitro*	—	—	↑Apoptotic cell death, arrested cells in the S-phase cell cycle, ROS, Bax, caspase-3 and caspase-9, PARP, ↓growth and migration, Bcl-2, MGMT, Wnt3a, *β*-catenin, AKT, NF-*κ*B	[90]

Hepatocellular carcinoma	H22 and HepG2 cells, *in vitro and in vivo*	—	—	↑LC3 II/I, autophagy, ↓cell proliferation, migration, and invasion, GM-CSF and G-CSF, PD-L1, p62, TNF-*α*, IL-6, and IL-17A, NF-*κ*B	[122]

Intrahepatic cholangiocarcinoma	ICC cells, *in vitro and in vivo*			↑Cell cycle arrest at G1 phase, ferroptosis, apoptosis, ↓cell proliferation and survival, invasion, and NF-*κ*B	[102]

Arrows (↑ and ↓) show an increase and decrease in the obtained variables, respectively. PYD: PYRIN domain; ASC: caspase recruitment domain; NLRP3: NLR family pyrin domain containing 3; 5-HT: 5-hydroxytryptamine; DA: dopamine; NE: neutrophil elastase; EGRF: epidermal growth factor receptor; ERF: ETS2 repressor factor; PARP: poly(ADP-ribose) polymerase; Caspase: cysteine-aspartic acid protease; MEK: mitogen-activated extracellular signal-regulated kinase; Bax: BcL2-associated X protein; Bcl-2: B-cell lymphoma; p53: tumor protein p53; p65: transcription factor p65; ER stress: endoplasmic reticulum stress; LC3-II: microtubule-associated protein 1A/1B-light chain 3; p62: ubiquitin-binding protein p62; AKT: protein kinase B; mTOR: mammalian (or mechanistic) target of rapamycin; p38: the serine/threonine kinase p38; MAPK: mitogen-activated protein kinases; ERK: extracellular signal-regulated kinase; MMP2 and MMP9: matrix metalloproteinase-2 and metalloproteinase-9; PI3K: phosphoinositide 3-kinase; Beclin1: protein which regulated autophagy; IL-2: interleukin-2; MFN1: mitofusin-1; Bcl-XL: B-cell lymphoma-extra-large; JAK/STAT: Janus kinase/signal transducers and activators of transcription; ROS: reactive oxygen species; NGFR: nerve growth factor receptor; pRb1: proline rich protein BstNI subfamily 1; CDK4: cyclin-dependent kinase 4; IFN-*γ*: interferon-gamma;; E-cadherin: epithelial cadherin; EMT: epithelial-mesenchymal transition; Bid: biotin acceptor domain, BH3 interacting domain death agonist; VEGF: vascular endothelial growth factor; TNF-*α*: tumor necrosis factor alpha; TNF-*β*: tumor necrosis factor beta; CSC: cancer stem cell; CAT level: chloramphenicol acetyltransferase level; BNIP3: BCL2 interacting protein 3; FIS1: mitochondrial fission 1 protein; TFAM: mitochondrial transcription factor A; BI6C9: bid inhibitor; SLC1A5: solute-linked carrier family A1 member 5; NRF2: nuclear factor erythroid 2 (NF-E2)-related factor 2; GPX4: glutathione peroxidase 4; Atg 5, 7, and 12: autophagy protein 5, 7, and 12; AMPK: AMP-activated protein kinase; ATM: ataxia telangiectasia mutated; HR: hormone receptor; NHEJ: nonhomologous end-joining; DDR: DNA damage response; NF-*κ*B: nuclear factor-*κ*B; GSH: glutathione.

**Table 5:** Immunotherapeutic role of quercetin against different cancers

Cancer type	Name of the compound	Test system	Dose/concentration (R/A)	IC_50_	Results/possible mechanism	References
Colorectal cancer	CD-PEG-FA.Rg3.QTN	CT26, HCT116, *in vitro* female BALB/c and nude mice (six to eight-week), *in vivo*	—	32 and 30 *μ*mol/L, respectively	↑ICD, ROS, blood circulation, cytotoxicity, apoptosis, BAX, caspase 9, and caspase 3, effector T cells, antiproliferative, ↓immunosuppressive TME, Bcl-2, CD4+ or CD8+ T cells	[181]

Microsatellite-stable colorectal cancer	QA-M	Colorectal cancer cell, *in vitro*	—	—	↑ICD, toxicity, immune response, memory tumor surveillance, ↓tumor growth; immunosuppressive TME	[187]

Breast cancer	Quercetin	MCF-10A, MCF-10AT, MCF-7 and MDA-MB-231, *in vitro*	5 *μ*M		↑Apoptosis, differentiation of *γδ* T cells into the V*δ*2 T cell subpopulation, IFN*γ*-R, p-JAK2 and p-STAT1, ↓PD-L1	[179]

Arrows (↑ and ↓) show an increase and decrease in the obtained variables, respectively. ICD; immunogenic cell death; TME: tumor microenvironment; Q: quercetin; A: alantolactone.

**Table 6:** Data of clinical trials in various cancers

Diseases/related effects	Gender (number of samples)	Dose and treatment duration	Biological activities/mechanisms	Side effects	References
Prostate cancer	Male, *n* = 31	1 gram of GTE (830 mg of GTP) with 800 mg of Q (GT + Q) for four weeks before prostatectomy	↑Bioavailability, EGC levels in urine and plasma, accumulation in plasma, urine, and prostate tissue, ↓methylation activity, liver toxicity	—	[192]

Ovarian cancer, hepatoma	11 patients (23–78 years)	Infusion at escalating doses initially at 3-week intervals. The first dose level was 60 mg/m^2^; at the 10th dose level of 1700 mg/m^2^	↓Lymphocyte tyrosine kinase activity, the serum alpha-fetoprotein	—	[193]

Cancer	Healthy male subjects aged between 33 and 64 years, *n* = 4	30 mg per day for 14 days	↑Antitumor activity, ↓TIMP-1	Aggressive disease and poor prognosis in patients with certain malignancies	[194]

Oxidative DNA damage.	114 females and 54 males, aged 18–45 years.	97 mg quercetin and 16 mg ascorbic acid a day	↑Plasma concentrations of quercetin, ascorbic acid, and TEAC, oxidative DNA damage	—	[195]

Polycystic ovary syndrome	Women, (*n* = 72)	500 mg of Quercetin for 40 days	↓LH, TNF-*α*, IL-6, inflammatory	—	[196]

Familial adenomatous polyposis	Five FAP patients with prior colectomy (4 with retained rectum and 1 with an ileal anal pouch)	Curcumin 480 mg and quercetin 20 mg orally 3 times a day.	↓The number and size of ileal and rectal adenomas	Nausea and sour taste	[197]

Arrows (↑ and ↓) show an increase and decrease in the obtained variables, respectively. EGC: epigallocatechin; TIMP-1: tissue inhibitor of metalloproteinases 1; TEAC: Trolox equivalent antioxidant capacity; IL-6: interleukin-6; GTE: green tea extract; GTP: green tea polyphenol.

## Data Availability

No data were used to support this study.
